# Impact of diabetes on outcome in critical limb ischemia with tissue loss: a large-scaled routine data analysis

**DOI:** 10.1186/s12933-017-0524-8

**Published:** 2017-04-04

**Authors:** Eva Freisinger, Nasser M. Malyar, Holger Reinecke, Holger Lawall

**Affiliations:** 1 Division of Vascular Medicine, Department of Cardiovascular Medicine, University Hospital Muenster, Albert Schweitzer Campus 1, A1, 48149 Muenster, Germany; 2 Praxis für Herzkreislauferkrankungen und Akademie für Gefaeßkrankheiten, Ettlingen, Germany

**Keywords:** Critical limb ischemia, Diabetes, Outcome, Epidemiology, Routine-data analysis

## Abstract

**Background:**

Patients with diabetes concomitant to critical limb ischemia (CLI) represent a sub-group at particular risk. Objective of this analysis is to evaluate the actual impact of diabetes on treatment, outcome, and costs in a real-world scenario in Germany.

**Methods:**

We obtained routine-data on 15,332 patients with CLI with tissue loss from the largest German health insurance, BARMER GEK from 2009 to 2011, including a follow-up until 2013. Patient data were analyzed regarding co-diagnosis with diabetes with respect to risk profiles, treatment strategy, in-hospital and long-term outcome including costs.

**Results:**

Diabetic patients received less overall revascularizations in Rutherford grades 5 and 6 (Rutherford grade 5: 45.0 vs. 55.5%; Rutherford grade 6: 46.5 vs. 51.8; p < 0.001) and less vascular surgery (Rutherford grade 5: 13.4 vs. 23.4; Rutherford grade 6: 19.7 vs. 29.6; p < 0.001), however more often endovascular revascularization in Rutherford grade 6 (31.0 vs. 28.1; p = 0.004) compared to non-diabetic patients. Diabetes was associated with a higher observed ratio of infections (35.3 vs. 23.5% Rutherford grade 5; 44.3 vs. 27.4% Rutherford grade 6; p < 0.001) and in-hospital amputations (13.0 vs. 7.3% Rutherford grade 5; 47.5 vs. 36.7% Ruth6; p < 0.001). Diabetes further increased the risk for amputation during follow-up [Rutherford grade 5: HR 1.51 (1.38–1.67); Rutherford grade 6: HR 1.33 (1.25–1.41); p < 0.001], but not for death.

**Conclusions:**

Diabetes increases markedly the risk of amputation attended by higher costs in CLI patients with tissue loss (OR 1.67 at Rutherford 5, OR 1.53 at Rutherford 6; p < 0.001), but is associated with lower revascularizations. However, in Rutherford grades 5 and 6, concomitant diabetes does not further worsen the overall poor survival.

**Electronic supplementary material:**

The online version of this article (doi:10.1186/s12933-017-0524-8) contains supplementary material, which is available to authorized users.

## Background

Critical limb ischemia (CLI), the chronic end-stage condition of lower extremity peripheral artery disease (PAD), is defined by clinical symptoms such as rest pain and/or ischemic lesions (ulcer or gangrene) objectively attributable to PAD [1, 2]. Patients with CLI are at high risk of limb loss: recent German nationwide data report an annual major amputation rate of 10% and a minor amputation rate of 15.5% per year in patients with ischemic lesions present (Rutherford grade 5 and 6) [3]. Further, CLI is an independent risk factor for cardiovascular events and death [4–6].

Diabetes mellitus is an important risk factor of arteriosclerotic diseases and has been reported to increase the risk of PAD by an Odds Ratio 1.68 (95% CI 1.53–1.84; p < 0.0001) in a global meta-analysis [7] and the relative risk for PAD has been shown to further increase with increasing duration of the diabetic status [8]. Accordingly, diabetes is frequently prevalent in patients with PAD and has been co-diagnosed in ∼35% of all in-patient PAD cases in Germany [3]. PAD patients with concurrent diabetes mellitus represent a subgroup at particular high risk for adverse outcome [9]. Co-morbidity with diabetes is particularly associated with (Mönckeberg’s) medial sclerosis to become manifest in the arteries below-the-knee [10–12]. Thus, diabetes ratio has been reported ∼80% in CLI patient with infrainguinal TransAtlantic Inter-Society Consensus (TASC) II C/D lesions [13], compared to ∼40% in overall CLI [3]. These often complex and highly calcified infrainguinal lesions are difficult to approach for both, surgical and endovascular revascularization procedures. Therefore, these are associated with restricted long-term patency and unfavorable outcome and the choice of the right treatment strategy in these patients remains challenging [4, 7]. Furthermore, diabetes and therewith attended conditions, such as polyneuropathy, foot malformation, or immunosuppression promote the development of ulcers and impaired wound healing [11], which again increase the risk of limb loss [6, 14–16]. Moreover, the presence of a diabetic foot syndrome is associated with a notable increase of minor amputations [17]. As a consequence, high rates of primary amputations are common in patients with CLI, particularly in those with diabetes [18, 19].

However, hitherto existing data on the impact of diabetes on patients with CLI are mainly small-sized sub-analyses of pre-selected patient populations. By reason of a lack of large-sized data on rather non-selected patients, conclusions on diabetic CLI patients with tissue loss are drawn from extrapolations of small-sized highly selected patient cohorts, leading to conflicting results [20].

Objective of our large-scaled routine data analysis is to investigate the recent health care situation of CLI patients with diabetes in terms of treatment strategies, complications and impact on outcome parameters as well as health expenditures in a real-world scenario.

## Methods

We obtained data on 15,332 patients hospitalized with the primary diagnosis of lower limb PAD at the stage of critical limb ischemia with tissue loss (CLI; ICD-10 codes I70.23–I70.24) in the time period between January 1st, 2009 and December 31st, 2011, including a follow-up until 2013 (median follow-up 677 days in Rutherford grade 5, and 577 days in Rutherford grade 6). Data were provided by the BARMER GEK as the largest public health insurance in Germany, comprising >8 million insured respective ∼10% of the German population.

Briefly, all in- and outpatient diagnostic and procedural data are encoded conformable to the diagnosis and procedure- related reimbursement system (German Diagnosis Related Groups, G-DRG system) as required by the mandatory coding guidelines and transferred to the health insurance for remuneration (in detail see [4]). These data are electronically verified and inspected by the personnel of the German Health Insurance Medical Service (MDK; Medizinischer Dienst der Krankenversicherung) in about 20% of cases.

We performed a retrospective analysis on all in- and outpatient diagnostic and procedural data of CLI patients classified in the Rutherford grades 5 (Rutherford grade 5; ICD-10 I70.23; n = 6916) and 6 (Rutherford grade 6; ICD-10 I70.24; n = 8416) (Additional file 1: Table S1). Patients with ischemic rest pain (Rutherford grade 4), that are also commonly recognized as CLI, were not included in the analysis, since particularly in diabetic patients rest pain may not be dependably ascertainable due to diabetic polyneuropathy. In contrast, tissue loss (Rutherford grades 5 and 6) is a much better objectifiable parameter. Within these Rutherford grades, we identified the patient subgroup with the co-diagnosis of diabetes mellitus (IDC-10 E10*, E11*) to be compared with the subgroup without encoded diabetes mellitus. We analyzed these in-hospital cases with respect to baseline characteristics such as age, sex, and the further co-diagnoses hypertension (ICD-10 I10-15*), obesity (ICD-10 E66), dyslipidemia (ICD-10 E78*), smoking (ICD-10 F17*), chronic kidney disease (ICD-10 N18*; CKD), coronary artery disease (ICD-10 I25*; CAD), chronic heart failure (ICD-10 I50*; CHF), and malignancies (ICD-10 C*). Further, we analyzed the encoded procedures during the index hospitalization: angiography (OPS 3-605, 3-607), any revascularization (OPS 5-380*, 5-381*, 5-383*, 5-386*, 5-388*, 5-393*, 5-395*, 8-836*, 8-84*), endovascular revascularization (EVR; OPS 8-836*, 8-84*), surgical revascularization (OPS 5-380*, 5-381*, 5-383*, 5-386*, 5-388*, 5-393*, 5-395*), thrombendartherectomy (TEA; OPS 5-381*), peripheral bypass surgery (OPS 5-393*). We evaluated the in-hospital complications acute renal failure (ICD-10 N17*), acute myocardial infarction (ICD-10 I21*), ischemic stroke (ICD-10 I63*), infection (ICD-10 A30-49*), and sepsis (ICD-10 B95-99*) as well as the in-hospital amputation (OPS 5-864*, 5-865*, 5-866*) and in-hospital mortality. A detailed listing on the diagnosis and procedural defining codes is presented in Additional file 1: Table S1.

### Statistics

Data on CLI patients were divided into four subgroups according to clinical stage (Rutherford grade 5/6) and diabetes status (DM/non-DM) for further analysis. Categorical variables are presented as absolute numbers (n) and percentages (%) of the total numbers for each subgroup; statistical comparisons for these were made by the Chi square test. The predictive value of baseline parameters [age, sex, hypertension, obesity, dyslipidemia, smoking, chronic kidney disease, coronary artery disease, acute myocardial infarction, chronic heart failure, and malignancies (as defined above)] concerning in-hospital mortality and amputation were tested by Binary logistic regression models; results were displayed as Odds Ratio (OR) with 95% confidence intervals (CI). Long-term amputation and long-term mortality were tested by multivariable cox regression models; results were displayed as hazard rate ratios (HR) with 95% CI, and cumulative event curves. To estimate the potential effect of variables to be adjusted for, that are not only risk factors but also sequences of PAD and diabetes (e.g. CAD, CKD, and CHF), additional Cox regression models with and without these variables has been performed (Additional file 2: Table S2).

We further provide an ANOVA-based costing analysis, itemized as in-hospital costs per index case, and aggregated in-hospital consequential costs including rehab expenses and presented as mean ± standard deviation (SD). All tests were performed two-sided, and p values of <0.05 were considered statistically significant.

## Results

### Baseline characteristics

We analyzed 15,332 patients with critical limb ischemia and tissue loss (CLI), there of 6916 (45.1%) at Rutherford grade 5 and 8416 patients at Rutherford category 6. The ratio of patients with diabetes was 44.3% in Rutherford grade 5 and 48.8% in Rutherford grade 6. Baseline characteristics including co-morbidities of all patients and of the sub-groups are presented in Table 1. In Rutherford grade 5 and Rutherford grade 6, patients with diabetes compared to non-diabetic patients (non-DM) were more likely to be male and to have higher frequency of hypertension, obesity, dyslipidemia, chronic kidney disease, coronary artery disease (CAD) and chronic heart failure (CHF, all p < 0.01). However, diabetic CLI patients were younger and less often smokers compared to patients without diabetes (p < 0.001, Table 1).

**Table 1:** Baseline characteristics and co-morbidities

	Rutherford grade 5				Rutherford grade 6			
	DM+	DM−	All	P	DM+	DM−	All	P
Patients, n (%)	3061 (44.3)	3855 (55.7)	6916 (100.0)		4108 (48.8)	4308 (51.2)	8416 (100.0)	
Male sex (%)	1795 (58.6)	1708 (44.3)	3503 (50.7)	<*0.001*	2551 (62.1)	2148 (49.9)	4699 (55.8)	<*0.001*
Age, mean ± SD	73.8 ± 10.8	77.0 ± 11.2	75.6 ± 11.1	<*0.001*	73.3 ± 11.2	76.5 ± 12.1	74.9 ± 11.8	<*0.001*
Hypertension (%)	2172 (71.0)	2514 (65.2)	4686 (67.8)	<*0.001*	2860 (69.9)	2577 (59.8)	5437 (64.6)	<*0.001*
Obesity (%)	361 (11.8)	186 (4.8)	547 (7.9)	<*0.001*	427 (10.4)	178 (4.1)	605 (7.2)	<*0.001*
Dyslipidemia (%)	834 (27.2)	836 (21.7)	1670 (24.1)	<*0.001*	922 (22.4)	752 (17.5)	1674 (19.9)	<*0.001*
Smoking (%)	140 (4.6)	293 (7.6)	433 (6.3)	<*0.001*	180 (4.4)	328 (7.6)	508 (6.0)	<*0.001*
CKD (%)	1179 (38.5)	960 (24.9)	2139 (30.9)	<*0.001*	1637 (39.8)	1160 (26.9)	2797 (33.2)	<*0.001*
CAD (%)	884 (28.9)	851 (22.1)	1735 (25.1)	<*0.001*	1218 (29.6)	974 (22.6)	2192 (26.0)	<*0.001*
CHF (%)	512 (16.7)	551 (14.3)	1063 (15.4)	*0.005*	766 (18.6)	704 (16.3)	1470 (17.5)	*0.005*
Malignancies (%)	55 (1.8)	87 (2.3)	142 (2.1)	0.180	103 (2.5)	137 (3.2)	240 (2.9)	0.064

Characteristics and co-diagnoses of Rutherford grade 5 and Rutherford grade 6 patients with and without diabetes (DM+/DM−). Data are given as patient numbers and percentages related to the respective subgroup. Statistical significance was tested via contingency table, p values <0.05 are considered significant

Italic values are statistically significant

*DM* diabetes mellitus; *CKD* chronic kidney disease; *CAD* coronary artery disease; *CHF* chronic heart failure

### Treatment

**Fig. 1:**
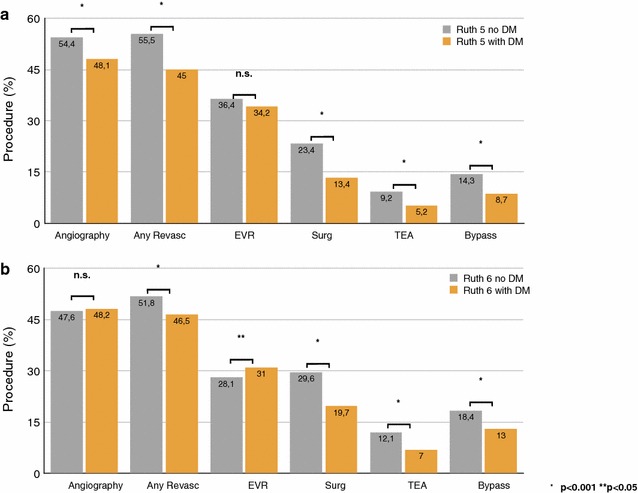
Treatment procedures related to diabetes status at Rutherford grade 5 and 6. Treatment procedures for angiography, overall revascularization (any revasc), endovascular revascularization (EVR), surgery (surg), thrombendartherectomy (TEA), and peripheral bypass in patients at Rutherford grade 5 (**a**) and Rutherford grade 6 (**b**) are given as percentages among patient sub-groups with diabetes (DM; *orange bars*) and without (*grey bars*). Differences between DM and non-DM sub-groups are considered significant for p values <0.05

In CLI patients at Rutherford grade 5, (diagnostic) invasive angiography was performed in 51.6% (n = 3567) patients, and the ratio of overall revascularization procedures (any revascularization) was 50.9% (n = 3518) (Table 2). Endovascular revascularization (EVR) has been performed in 35.4% (n = 2450) patients, and vascular surgery [peripheral bypass and/or thrombendartherectomy (TEA)] was performed in 19% (n = 1312) patients (Fig. 1). Comparison between diabetic and non-diabetic patients at Rutherford grade 5 showed patients with DM to receive less angiography (48.1 vs. 54.4; −11.6%), overall revascularization (45.0 vs. 55.5%; −18.9%), surgery (13.4 vs. 23.4; −42.7%), TEA (5.2 vs. 9.2; −43.5%), and bypass procedures (8.7 vs. 14.3; −39.2%; all p < 0.001) (Table 2). The ratio of EVR did not significantly differ between diabetic and non-diabetic patients at Rutherford grade 5 (34.2 vs. 36.4; p = 0.059). In Rutherford grade 6, the ratio of angiography did not differ significantly between diabetic and non-diabetic patients. However, overall revascularization rate was lower in diabetic compared to non-diabetic patients (46.5 vs. 51.8; −10.2%; p < 0.001). Rutherford grade 6 patients with DM received more often EVR compared to non-DM patients (31.0 vs. 28.1; +10.3%; p = 0.004), but less often surgical revascularization (overall surgery 19.7 vs. 29.6; −33.4%; TEA 7.0 vs. 12.1; −42.1%; bypass 13.0 vs. 18.4; −29.3%; all p < 0.001) (Fig. 1; Table 2).

**Table 2:** Treatment, in-hospital complications and outcome

	Rutherford grade 5				Rutherford grade 6			
	DM+	DM−	All	P	DM+	DM−	All	P
Patients, n (%)	3061 (44.3)	3855 (55.7)	6916 (100.0)		4108 (48.8)	4308 (51.2)	8416 (100.0)	
Angiography (%)	1471 (48.1)	2096 (54.4)	3567 (51.6)	<*0.001*	1982 (48.2)	2050 (47.6)	4032 (47.9)	0.544
Any revascularization (%)	1377 (45.0)	2141 (55.5)	3518 (50.9)	<*0.001*	1909 (46.5)	2231 (51.8)	4140 (49.2)	<*0.001*
EVR (%)	1047 (34.2)	1403 (36.4)	2450 (35.4)	*0.059*	1272 (31.0)	1209 (28.1)	2481 (29.5)	*0.004*
Surgery (%)	409 (13.4)	903 (23.4)	1312 (19.0)	<*0.001*	809 (19.7)	1274 (29.6)	2083 (24.8)	<*0.001*
TEA (% of all) (% of surgery)	160 (5.2) *(39.1)*	354 (9.2) *(39.2)*	514 (7.4) *(39.2)*	<*0.001*	287 (7.0) *(35.5)*	520 (12.1) *(40.8)*	807 (9.6) *(38.7)*	<*0.001*
Bypass (% of all) (% of surgery)	266 (8.7) *(65.0)*	550 (14.3) *(60.9)*	816 (11.8) *(62.2)*	<*0.001*	532 (13.0) *(65.8)*	794 (18.4) *(62.3)*	1326 (15.8) *(63.7)*	<*0.001*
ARF (%)	61 (2.0)	66 (1.7)	127 (1.8)	0.388	115 (2.8)	120 (2.8)	235 (2.8)	0.969
AMI (%)	22 (0.7)	36 (0.9)	58 (0.8)	0.330	80 (1.9)	67 (1.6)	147 (1.7)	0.170
Ischemic stroke (%)	14 (0.5)	15 (0.4)	29 (0.4)	0.663	31 (0.8)	32 (0.7)	63 (0.7)	0.950
Infection (%)	1082 (35.3)	905 (23.5)	1987 (28.7)	<*0.001*	1821 (44.3)	1180 (27.4)	3001 (35.7)	<*0.001*
Sepsis (%)	150 (4.9)	173 (4.5)	323 (4.7)	0.419	277 (6.7)	214 (5.0)	491 (5.8)	*0.001*
In-hospital amputation (%)	399 (13.0)	280 (7.3)	679 (9.8)	<*0.001*	1952 (47.5)	1579 (36.7)	3531 (42.0)	<*0.001*
In-hospital death (%)	80 (2.6)	154 (4.0)	234 (3.4)	*0.002*	300 (7.3)	401 (9.3)	701 (8.3)	*0.001*

Treatment, in-hospital complications and outcome of Rutherford grade 5 and Rutherford grade 6 patients with and without diabetes (DM+/DM−). Data are given as patient numbers and percentages related to the respective subgroup. Statistical significance was tested via contingency table, p values <0.05 are considered significant

Italic values are statistically significant

*DM* diabetes mellitus; *EVR* endocascular revascularization; *TEA* thrombartherectomy; *ARF* acute renal failure; *AMI* acute myocardial infarction

### Complications and in-hospital outcome

Most frequent complication was infection with overall 28.7% in Rutherford grade 5 and 35.7% in Rutherford grade 6. Diabetes strongly increased the ratio of infections in Rutherford grade 5 (35.3 vs. 23.5 non-DM; p < 0.001), and in Rutherford grade 6 (44.3 vs. 27.4; p < 0.001) (Additional file 3: Figure S1). The occurrence of sepsis did not differ between DM and non-DM patients at Rutherford grade 5 (4.9 vs. 4.5%; p = n.s.) but was increased in DM patients at Rutherford grade 6 (6.7% vs. 5.0%; p = 0.001). There was no significant differences between DM and non-DM patients in the occurrence of acute renal failure (ARF; Rutherford grade 5: 2.0 vs. 1.7; Rutherford grade 6: 2.8 vs. 2.8), acute myocardial infarction (AMI; Rutherford grade 5: 0.7 vs 0.9; Rutherford grade 6: 1.9 vs. 1.6), and ischemic stroke (Rutherford grade 5: 0.5 vs. 0.4; Rutherford grade 6: 0.8 vs 0.7; all p = n.s.) (Table 2). The observed ratio of in-hospital amputations in Rutherford grade 5 was higher in patients with diabetes compared to non-DM patients (13.0 vs. 7.3; p < 0.001). In Rutherford grade 6, in-hospital amputations further increased to 47.5% in DM vs. 36.7% in non-DM patients (p < 0.001) (Additional file 3: Figure S1, Table 2). The observed in-hospital mortality of DM patients was lower with 2.6% compared to 4.0% in non-DM patients in Rutherford grade 5 (p = 0.002), and 7.3% vs. 9.3% in Rutherford grade 6 (p = 0.001). Adjusting for baseline risk factors in a binary logistic regression model showed diabetes be associated with decreased in-hospital mortality by an Odds Ratio of 0.57 for Rutherford grade 5, and 0.7 for Rutherford grade 6; in-hospital amputation was significantly increased by concomitant diabetes (Odds Ratio 1.67 at Rutherford 5, 1.53 at Rutherford 6; all p < 0.001; see Additional file 4: Table S3).

### Long-term outcome

**Fig. 2:**
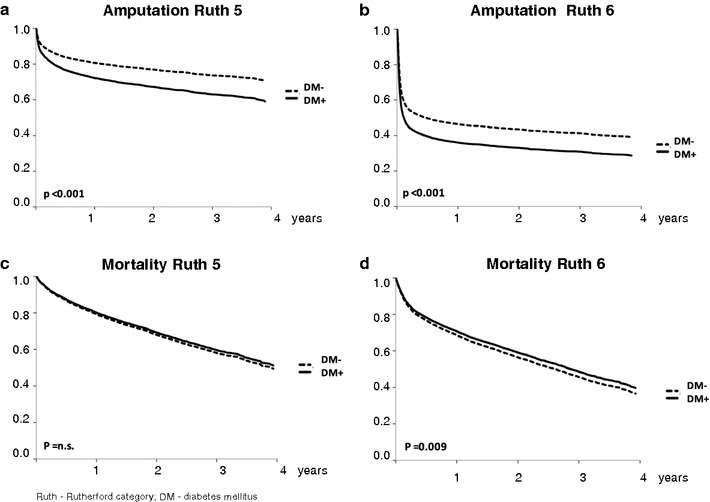
Cox regression analysis of amputations and long-term mortality related to Rutherford grade and diabetes status. **a** *–* **d** show Cox regression analyses for the end-points amputation and mortality during the 4-year follow-up period within Rutherford grade 5 and Rutherford grade 6. Amputation rate is significantly higher in patients with diabetes (DM; *continuous line*) compared to non-DM patients (*dashed line*) in Rutherford grade 5 (**a**) and Rutherford grade 6 (**b**). Mortality does not significantly differ between DM and non-DM patients in Rutherford grade 5 (**c**) and is slightly lower in DM compared to non-DM patients in Rutherford grade 6 (**d**). p values <0.05 are regarded significant

In the multivariate Cox regression analysis, diabetes increased the risk for amputation during the follow-up period by a Hazard Ratio (HR) of 1.51 (95% CI 1.38–1.67; p < 0.001) in Rutherford grade 5 and by a HR of 1.33 (95% CI 1.25–1.41; p < 0.001) in Rutherford grade 6 (Table 3). Therefore, the adjusted amputation rate at 4 years post index-hospitalization was 41% in diabetic compared to 29% in non-diabetic patients in Rutherford grade 5, and 71 vs. 61% in Rutherford grade 6 patients, respectively (Fig. 2). Further, the risk for amputation during long-term follow-up was increased by male gender (Rutherford grade 5: HR 1.48; p < 0.001; Rutherford grade 6: HR 1.27; p < 0.001), presence of CKD (only Rutherford grade 5: HR 1.33; p < 0.001) and heart failure (only Rutherford grade 6: HR 1.10; p = 0.012). Protective factors in Rutherford grade 5 were age (HR 0.99; p = 0.01) and obesity (HR 0.79; p = 0.007), whereas in Rutherford grade 6 hypertension (HR 0.93; p = 0.017), dyslipidemia (HR 0.85; p < 0.001), and smoking (HR 0.81; p < 0.001) turned out to decrease the amputation risk during follow-up (Table 3).

**Table 3:** Cox regression analysis on long-term outcome (mortality and amputation)

	Rutherford grade 5				Rutherford grade 6			
	Mortality		Amputation		Mortality		Amputation	
	HR (95% CI)	P	HR (95% CI)	P	HR (95% CI)	P	HR (95% CI)	P
Age	1.05 (1.04–1.05)	<*0.001*	0.99 (0.99–1.00)	*0.010*	1.05 (1.05–1.06)	<*0.001*	1.00 (1.00–1.00)	0.951
Male gender	1.13 (1.04–1.23)	*0.003*	1.48 (1.34–1.63)	<*0.001*	1.14 (1.07–1.22)	<*0.001*	1.27 (1.20–1.35)	<*0.001*
Hypertension	0.80 (0.74–0.86)	<*0.001*	0.91 (0.82–1.01)	0.066	0.82 (0.77–0.88)	<*0.001*	0.93 (0.87–0.99)	*0.017*
Obesity	0.68 (0.57–0.82)	<*0.001*	0.79 (0.66–0.94)	*0.007*	0.81 (0.70–0.93)	*0.002*	0.96 (0.87–1.07)	0.502
Dyslipidemia	0.75 (0.68–0.82)	<*0.001*	0.96 (0.86–1.07)	0.442	0.76 (0.70–0.83)	<*0.001*	0.85 (0.79–0.92)	<*0.001*
Smoking	0.99 (0.81–1.20)	0.896	0.91 (0.75–1.11)	0.372	0.98 (0.84–1.14)	0.804	0.81 (0.72–0.92)	*0.001*
Diabetes	0.95 (0.88–1.03)	0.222	1.51 (1.38–1.67)	<*0.001*	0.92 (0.86–0.98)	*0.009*	1.33 (1.25–1.41)	<*0.001*
CAD	1.20 (1.10–1.31)	<*0.001*	1.04 (0.93–1.16)	0.479	1.25 (1.17–1.34)	<*0.001*	0.98 (0.91–1.05)	0.514
CHF	1.63 (1.49–1.79)	<*0.001*	0.94 (0.82–1.08)	0.410	1.50 (1.39–1.61)	<*0.001*	1.10 (1.02–1.19)	*0.012*
CKD	1.55 (1.43–1.68)	<*0.001*	1.33 (1.20–1.47)	<*0.001*	1.38 (1.30–1.47)	<*0.001*	0.98 (0.92–1.05)	0.572
Malignancies	1.73 (1.38–2.16)	<*0.001*	0.97 (0.68–1.37)	0.855	1.93 (1.66–2.24)	<*0.001*	0.86 (0.72–1.03)	0.106

Cox regression analysis for the end-points mortality and amputation during the 4-year follow-up period in patients at Rutherford grade 5 and Rutherford grade 6. In the proportional hazards model, effect of included variables are presented as Hazard Ratio and corresponding confidence intervals, p values <0.05 are considered significant

Italic values are statistically significant

*HR* Hazard Ratio; *CI* confidence interval; *CAD* coronary artery disease; *CHF* chronic heart failure; *CKD* chronic kidney disease

In the multivariate Cox regression analysis, adjusted mortality was approx. 50% at 4 years follow-up with no significant difference between diabetic and non-diabetic patients at Rutherford grade 5, and in Rutherford grade 6 long-term mortality was 60% in patients with DM vs. 63% without DM (Fig. 2). Therefore, diabetes had no significant impact on adjusted long-term mortality in Rutherford grade 5 (HR 0.95; 95% CI 0.88–1.03; p = n.s.), and turned out slightly protective of long-term mortality in Rutherford grade 6 with a HR 0.92 (95% CI 0.86–0.98; p = 0.009) (Table 3). However, if the risk of long-term mortality was relevantly impacted by CAD, CKD, and CHF (Additional file 2: Table S2), showing the protective effect to diminish if omitted from the Cox regression. Further, male sex, age, and malignancies increased long-term mortality, whereas protective association with long-term mortality was found for hypertension, obesity, and dyslipidemia in Rutherford grades 5 and 6 (Table 3). No significant impact, besides for diabetes, was found for smoking.

### Treatment costs

Costs per case of the index hospitalization were about equal for diabetic patients with 5975 EUR compared to non-diabetic patients with 6058 EUR at PAD Rutherford grade 5, and 8860 EUR vs. 8080 EUR at Rutherford grade 6, respectively (Additional file 5: Figure S2). However, the accumulated costs of the subsequent in-patient treatments (including rehab) during the follow-up period were higher in PAD patients with diabetes compared to those without: 22,904 EUR in DM vs. 19,596 EUR in non-DM per-case in Rutherford grade 5, and 23,006 EUR vs. 19,204 EUR per-case in Rutherford grade 6, respectively (p < 0.01). Thus, subsequent in-patient costs for CLI patients with diabetes sum up to a total of 136,247,036 EUR (to be allotted to 7169 patients in the analysis) compared to 122,093,921 EUR (8163 patients) without diabetes.

## Discussion

We found concomitant diabetes in CLI patients with tissue loss to be associated with lower revascularization procedures and increased amputation rate during in-hospital and follow-up period. However, mortality rate was not further increased subject to diabetes status in this end-stage PAD patient subset.

### Characteristics of CLI Patients with and without diabetes

Diabetes is a common aggravating co-morbidity in patients with peripheral artery disease (PAD) and the diabetes ratio of 45–50% among CLI patients in our patient collective corresponds well with the nationwide diabetes ratio of 39.3% of in-patient PAD cases at Rutherford grade 5 and 6, as documented by the Research Data Centers of the Federal Bureau of Statistics and the statistical offices of the federal states (DESTATIS) [3]. CLI patients with diabetes are at average 3 years younger compared to non-diabetic patients at the same Rutherford grade. This trend conforms with other studies [11, 21], and likely reflects the progressing effect of diabetes on the arterial calcification and clinical severity of PAD. Further, diabetic CLI patients are shown higher ratio of obesity, dyslipidemia, or hypertension as common co-morbidities (“metabolic syndrome”), as well as higher ratio of chronic kidney disease or coronary artery disease as common sequelae of diabetes [22]. The lower smoking rate in diabetic CLI patients [21] might partially result of a selection bias, since smoking dramatically increases the risk for PAD [adjusted Odds Ratio 10.4 (3.8–28.3) compared to Odds Ratio 2.3 (1.4–3.8) for diabetes; p < 0.01; [23]), yet cannot be entirely explained; on the other hand, diabetic patients might benefit from frequent ambulatory consultations including educative and preventive measures eventually resulting in decreased rate of tobacco use. We observed a higher ratio of male patients in diabetic compared to non-diabetic CLI patients with tissue loss. In other population based studies, the global incidence of PAD has been reported to not relevantly differ between males and females (4.5 vs. 4.2%; [24]), however female patients get diagnosed ca. 3–4 years later yet present at higher stages of PAD [25, 26].

### Treatment in CLI Patients with and without diabetes

The current scientific opinion regarding therapeutic options for CLI highlights the indispensable need to reestablish a best possible blood perfusion of the affected limb, prevailing regardless of patient’s gender [1, 2]. Considering this, the application of (diagnostic) angiography and use of revascularization procedures in only about 50% of patients with CLI confirm a dramatic under-usage of guideline recommended therapy, as reported previously [4]. By comparison, patients with diabetes tend to receive less often angiography, overall revascularization and particularly surgical procedures. Physicians might be more cautious in the use of methods that require contrast medium, such as angiography or endovascular procedures, being concerned of worsened kidney function on the basis of an often concomitant chronic kidney disease. However, recent data substantiate this concern unfounded [27] and rated secondary in the face of a high risk for limb loss and death, particularly associated with non-healed wounds [6, 17, 19, 28].

### In-hospital and long-term outcome in CLI patients with and without diabetes: Amputation

The observed in-hospital amputation rates in diabetic PAD patients of more than 10% in Rutherford grade 5 (+78% increase compared to non-diabetic) and almost 50% in Rutherford grade 6 (+29% increase compared to non-diabetic) point out this diabetic CLI sub-population to be at particular high risk for limb loss. One reason for increased limb loss, apart from lower revascularization attempts in diabetic patients, may involve the increased risk of infections, as shown as the major complication with more than one-third in diabetic CLI patients at Rutherford grades 5 and 6. Binary logistic regression analysis adjusting for patient’s risk profiles substantiate diabetes to be independently associated with an increased risk for in-hospital amputation (Odds Ratio 1.67 in Rutherford 5, and 1.53 in Rutherford 6; p < 0.001). Cox regression analysis further confirms diabetes to independently increase the risk for amputation during a 4-year follow up (HR 1.51 in Rutherford grade 5, HR 1.33 in Rutherford grade 6). These numbers correspond well with other studies, showing diabetes to predict major amputation by HR 1.56 (major amputation 30.8% in diabetic vs. 20.4% in non-diabetic patients within 4 years) [19].

Further, Spreen et al. [19] found long-term mortality risk to be significantly increased by amputation, although it has to be considered that the patient collective of the underlying RCTs was selective and patient number was small (n = 281). A recent meta-analysis reported diabetes to be associated with an increased risk of mortality in CLI (Odds Ratio 2.38; 95% CI 1.22–4.63; p < 0.001) [20]. However, the studies in this meta-analysis (CLI ratio between 12.3 and 100%) show highly varied overall mortality rates ranging from 10.2 to 54.2%, reflecting the heterogeneity of these studies (only five studies were exclusively on CLI patients). Importantly, objective of the preponderant part of the included mainly small-sized studies was to compare specific treatment strategies in therefore highly selected patient cohorts, and a closer view reveals actually conflicting results on the impact of diabetes on CLI mortality [29, 30]. Moreover, studies including a higher percentage of patients at Rutherford grade 4 (rest pain), as also commonly recognized as CLI, may substantially differ from our results on CLI with tissue loss in terms of amputation rates and mortality risk. In fact, several authors have recently requestioned a redefinition of CLI grading up objective diagnostic criteria, or at least to regard Rutherford grade 4 and Rutherford grades 5–6 as two different entities by reason of differing risk profiles and prognoses [31–33].

### In-hospital and long-term outcome in CLI patients with and without diabetes: Mortality

Our large-scaled data on 15,332 CLI patients at Rutherford grades 5 and 6, reflecting the “real-world” situation, do not show increased mortality in the diabetic sub-group. In fact, despite lower revascularization and higher amputation rates in diabetic patients, observed in-hospital mortality is surprisingly higher in non-diabetic patients. This may mainly reflect a strong effect of the increased age in the non-diabetic sub-group, that may insufficiently represented in the binary logistic regression. In the long-term period, diabetes turns out to be only slightly protective of long-term mortality in Rutherford grade 6 by HR 0.92. Taking further the strong effect of the concomitant diagnoses coronary artery disease, chronic kidney disease, and chronic heart failure into account, that are indeed risk factors but on the other hand also sequences of PAD and diabetes, the impact of diabetes on long-term mortality becomes insignificant. It may be concluded, that end-stage PAD patients with tissue loss have such poor prognosis in terms of survival (50–60% at 4 years), that concomitant diabetes itself would not relevantly further increase mortality rates. This finding is paralleled by Humphries et al. [21] that did not show any further deteriorating effect of diabetes on in-hospital mortality of PAD patients with lower extremity ulcers on statewide data for California, USA. In face of the recent nationwide study by Agarwal et al. [34] the more frequently used endovascular treatment strategy in the diabetic sub-group might add to the improved survival. Further, the equalization of the survival trends in diabetic patients may hint to the benefits that accrue of the meanwhile comprehensively high therapy standards in the treatment of diabetes [6, 35–37]. However, these may add to the increased subsequent treatment costs of diabetic CLI patients [38].

### Strengths and limitations

There is an ongoing debate on the significance of the use of routine-data regarding its accuracy, detailedness and transferability to clinical evidence. DRG-based data are recorded for remuneration purpose and therefore, secondary analyses of these data addressing health care quality are limited by the lack of causal nexus. Further commonly acknowledged limitations of a retrospective study design, such as potential selection and information bias apply to our analysis. For instance, information on medication, ambulatory measures taken, or other metabolic factors that might have influenced outcome parameters [39] could not be assessed. However, integrity of the data-bases and reliability of the encoded data are extremely high, particularly by reason of statutory provisions and integrated monitoring systems. Large-scaled population based data allow a statement on the current health service in a real-world setting, pointing out important supply shortage, under-utilization and populations at particular risks for hard clinical end-points. The data provided by the BARMER GEK are of moderate detailedness and allow due to the patient-, but not case-based record, a solid statistical adjustment of co-variates. Further, data include a follow-up period of up to 4 years.

## Conclusion

Patients with CLI and concomitant diabetes receive less revascularization procedures compared to non-diabetic patients despite being at particular high risk for amputation. This reflects in increased subsequent in-hospital costs. However, diabetes was not associated with increased mortality of CLI patients with tissue loss. Further research is needed to identify the underlying reasons for the under-usage of revascularization procedures in diabetic CLI patients to improve limb salvage and reduce health expenditure.

## Additional files

**Additional file 1: Table S1.** ICD- and OPS codes of diagnoses and procedures.

**Additional file 2: Table S2.** Impact of Choice of Variables in multivariate Cox regression models on the potential “protective”role of diabetes on long-term Mortality.

**Additional file 3: Figure S1.** In-hospital complications in patients with vs. without diabetes at Rutherford grade 5 and 6. Complication rates for infection, sepsis, in-hospital amputation, and in-hospital death in patients at Rutherford grade 5 (panel A) and Rutherford grade 6 (panel B) are given as percentages among patient sub-groups with diabetes (DM; orange bars) and without (grey bars). Differences between DM and non-DM sub-groups are considered significant for p-values < 0.05.

**Additional file 4: Table S3.** Binary Logistic Regression Analysis of In-hospital Outcomes.

**Additional file 5: Figure S2.** Costing analysis related to Rutherford grade and diabetes status. Costs per case are given in EURO for the in-hospital period (bottom dark bars) and subsequent in-patient costs (upper light bars) in diabetic (DM; orange) and non-DM patients (grey) related to Rutherford grades. Data show about equal in-hospital costs for patients with and without diabetes at the same Rutherford grade, but increased subsequent in-patient costs in patients with DM compared to non-DM CLI patients. Costs are increasing with increasing Rutherford grade.

## Abbreviations

AMI: acute myocardial infarction
ARF: acute renal failure
CAD: coronary artery disease
CHF: chronic heart failure
CI: confidence interval
CKD: chronic kidney disease
CLI: critical limb ischemia
DESTATIS: Research Data Centers of the Federal Bureau of Statistics and the statistical offices of the federal states
DM: diabetes mellitus
DRG: German Diagnosis Related Groups
EVR: endovascular revascularization
HR: Hazard Ratio
MDK: Medizischer Dienst der Krankenversicherung
non-DM: no diabetes mellitus
OPS: Operationen und Prozedurenschlüssel
OR: Odds Ratio
PAD: peripheral artery disease
RCT: randomized controlled trial
SD: standard deviation
TASC: Transatlantic Inter-Society Consensus
TEA: thrombendartherectomy
