# Supplementary material for: Impact of diabetes on outcome in critical limb ischemia with tissue loss: a large-scaled routine data analysis
Source: Cardiovasc Diabetol. 2017 Apr 4;16:41. doi: 10.1186/s12933-017-0524-8 (PMC5379505; doi:10.1186/s12933-017-0524-8)
Supplement: Supplementary file 1 — Additional file 1: Table S1. ICD- and OPS codes of diagnoses and procedures. [file 12933_2017_524_MOESM1_ESM.docx]

**Supplementary Table S1: ICD- and OPS codes of diagnoses and procedures**

| **Diagnosis** | **ICD-10 GM** |
| --- | --- |
| Peripheral Artery Disease; Rutherford grade 5 | I70.23 |
| Peripheral Artery Disease; Rutherford grade 6 | I70.24 |
| Diabetes mellitus | E10*, E11* |
| Hypertension | I10-15* |
| Obesity | E66 |
| Dyslipidemia | E78* |
| Smoking | F17* |
| Chronic kidney disease | N18* |
| Coronary artery disease | I25* |
| Chronic heart failure | I50* |
| Malignancies | C* |
| Acute renal failure | N17* |
| Acute myocardial infarction | I21* |
| Ischemic stroke | I63* |
| Infection | A30-49* |
| Sepsis | B95-99* |
| Procedure | OPS-Code |
| Angiography | 3-605, 3-607 |
| Any revascularization | 5-380*, 5-381*, 5-383*, 5-386*, 5-388*, 5-393*, 5-395*, 8-836*, 8-84* |
| Endovascular revascularization | 8-836*, 8-84* |
| Surgery | 5-380*, 5-381*, 5-383*, 5-386*, 5-388*, 5-393*, 5-395* |
| Thrombendartherectomy | 5-381* |
| Bypass | 5-393* |
| Amputation | 5-864*, 5-865*, 5-866* |

ICD-10 GM - International Statistical Classification of Diseases and Related Health Problems German Modification version 10; OPS - Operation and Procedural Code. All procedures and interventions are encoded with according to the ICD10-GM and OPS system in purose of remuneration via the German DRG system. The * indicates that additional digits for specific descriptions of types and anatomic locations can/must be added. OPS indicates “Operationen und Prozedurenschlüssel”.
