# Supplementary material for: Impact of diabetes on outcome in critical limb ischemia with tissue loss: a large-scaled routine data analysis
Source: Cardiovasc Diabetol. 2017 Apr 4;16:41. doi: 10.1186/s12933-017-0524-8 (PMC5379505; doi:10.1186/s12933-017-0524-8)
Supplement: Supplementary file 2 — Additional file 2: Table S2. Impact of Choice of Variables in multivariate Cox regression models on the potential “protective”role of diabetes on long-term Mortality. [file 12933_2017_524_MOESM2_ESM.docx]

**Supplementary Table S2: Impact of Choice of Variables in multivariate Cox regression models on the potential „protective“ role of diabetes on long-term Mortality**.

| ***Cox Regression Model (Table 3, Figure 2)*** | | | |
| --- | --- | --- | --- |
| End-point long-term Mortality | | | |
| adjusted for variables: | *age, sex, hypertension, obesity, dyslipidemia, smoking, diabetes, malignancies, coronary artery disease, chronic kidney disease, chronic heart failure* | | |
| *Rutherford 5:* | *Diabetes HR 0.95* | *95% CI 0.88-1.03* | *p = n.s.* |
| *Rutherford 6:* | *Diabetes HR 0.92* | *95% CI 0.86-0.98* | *p=0.009* |
| ***Cox Regression Model 1 - adjusted solely for baseline atherosclerotic risk factors*** | | | |
| End-point long-term Mortality | | | |
| adjusted for variables: | *age, sex, hypertension, obesity, dyslipidemia, smoking, diabetes, malignancies* | | |
| *Rutherford 5:* | *Diabetes HR 1.039* | *95% CI 0.961-1.124* | *p= n.s.* |
| *Rutherford 6:* | *Diabetes HR 0.988* | *95% CI 0.928-1.051* | *p=n.s.* |
| ***Cox Regression Model 2 - adjusted solely for CV diseases and complications.*** | | | |
| End-point long-term Mortality | | | |
| adjusted for variables: | *coronary artery disease, chronic kidney disease, chronic heart failure, diabetes* | | |
| *Rutherford 5:* | *Diabetes HR 0.79* | *95% CI 0.729-0.85* | *p<0.001* |
| *Rutherford 6:* | *Diabetes HR 0.76* | *95% CI 0.715-0.808* | *p<0.001* |

*Model 1 shows that exclusion of the cardiovascular diseases diminishes the effect of diabetes on mortality, as this does not reach statistical significance in both Rutherford grades. The strength of the effect of cardiovascular diseases can be seen in Model 2, where baseline risk factors are excluded from the analysis.*
