# Supplementary figures and images for: Impact of diabetes on outcome in critical limb ischemia with tissue loss: a large-scaled routine data analysis
Source: Cardiovasc Diabetol. 2017 Apr 4;16:41. doi: 10.1186/s12933-017-0524-8 (PMC5379505; doi:10.1186/s12933-017-0524-8)

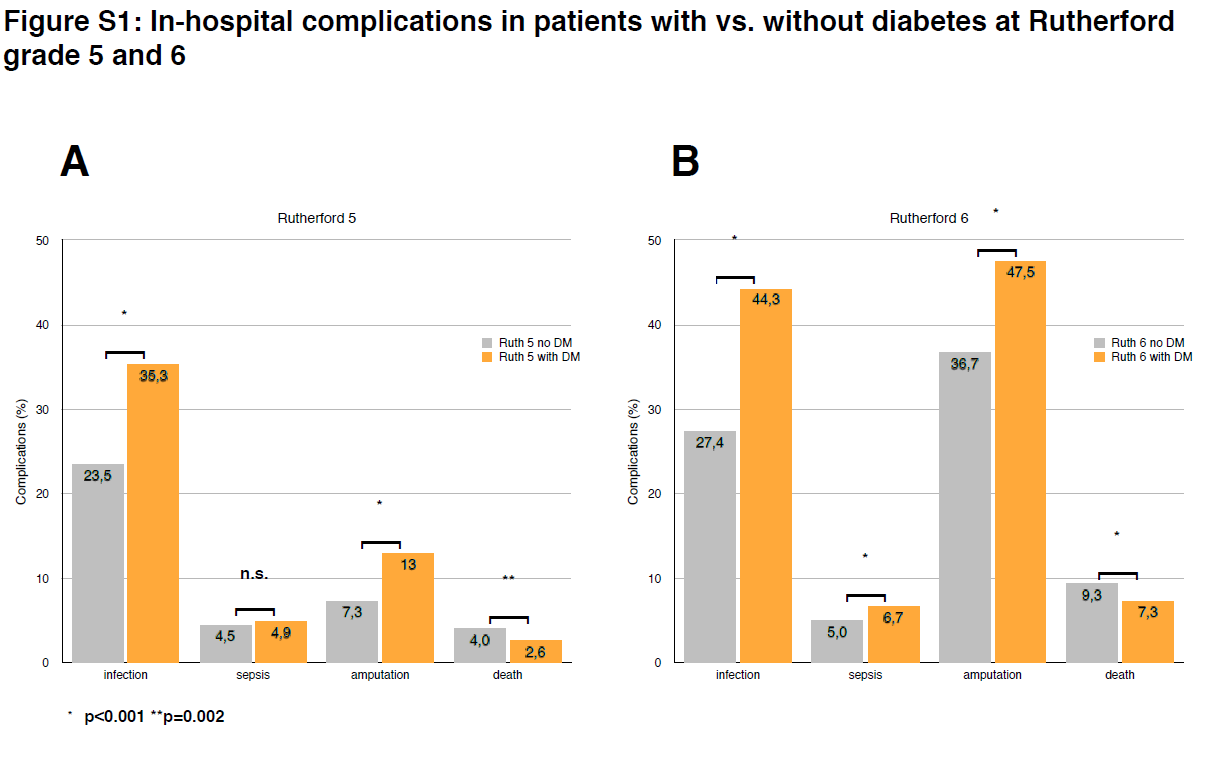

Supplement: Supplementary file 3 — Additional file 3: Figure S1. In-hospital complications in patients with vs. without diabetes at Rutherford grade 5 and 6. Complication rates for infection, sepsis, in-hospital amputation, and in-hospital death in patients at Rutherford grade 5 (panel A) and Rutherford grade 6 (panel B) are given as percentages among patient sub-groups with diabetes (DM; orange bars) and without (grey bars). Differences between DM and non-DM sub-groups are considered significant for p-values < 0.05. [file 12933_2017_524_MOESM3_ESM.docx]

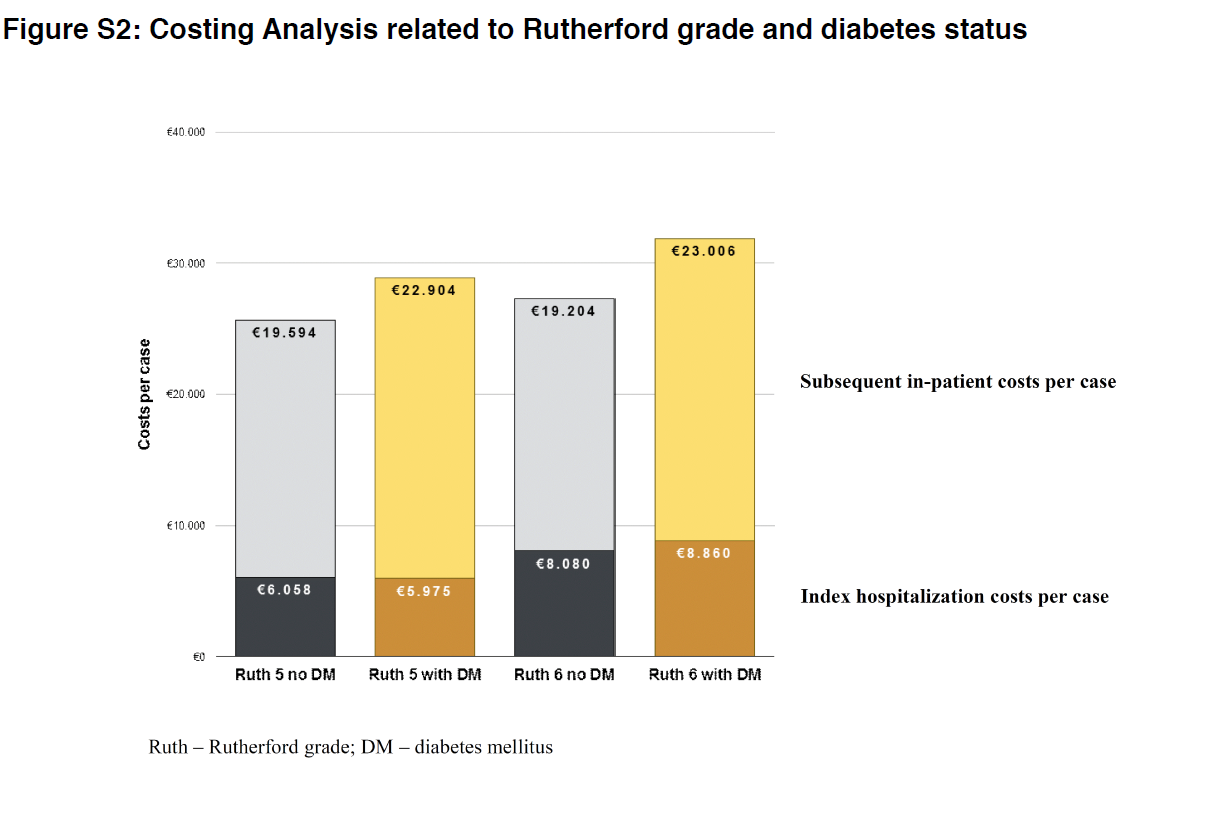

Supplement: Supplementary file 5 — Additional file 5: Figure S2. Costing analysis related to Rutherford grade and diabetes status. Costs per case are given in EURO for the in-hospital period (bottom dark bars) and subsequent in-patient costs (upper light bars) in diabetic (DM; orange) and non-DM patients (grey) related to Rutherford grades. Data show about equal in-hospital costs for patients with and without diabetes at the same Rutherford grade, but increased subsequent in-patient costs in patients with DM compared to non-DM CLI patients. Costs are increasing with increasing Rutherford grade. [file 12933_2017_524_MOESM5_ESM.docx]
