# Supplementary material for: Impact of diabetes on outcome in critical limb ischemia with tissue loss: a large-scaled routine data analysis
Source: Cardiovasc Diabetol. 2017 Apr 4;16:41. doi: 10.1186/s12933-017-0524-8 (PMC5379505; doi:10.1186/s12933-017-0524-8)
Supplement: Supplementary file 4 — Additional file 4: Table S3. Binary Logistic Regression Analysis of In-hospital Outcomes. [file 12933_2017_524_MOESM4_ESM.docx]

**Supplementary Table S3: Binary Logistic Regression Analysis of In-hospital Outcomes**

|  | **Rutherford grade 5** | | | | **Rutherford grade 6** | | | |
| --- | --- | --- | --- | --- | --- | --- | --- | --- |
|  | Mortality (n=234) | | Amputation (n=679) | | Mortality (n=701) | | Amputation (n=3,531) | |
|  | **OR (95% CI)** | **p** | **OR (95% CI)** | **p** | **OR (95% CI)** | **p** | **OR (95% CI)** | **p** |
| **Age** | 0.97 (0.97-0.98) | **< 0.001** | 0.97 (0.97-0.98) | **< 0.001** | 0.98 (0.98-0.98) | **< 0.001** | 0.99 (0.99-0.99) | **< 0.001** |
| **Male gender** | 0.63 (0.49-0.82) | **0.001** | 1.3 (1.1-1.53) | **0.002** | 0.7 (0.6-0.82) | **< 0.001** | 1.29 (1.18-1.42) | **< 0.001** |
| **Hypertension** | 0.52 (0.39-0.68) | **< 0.001** | 0.87 (0.73-1.03) | 0.110 | 0.62 (0.52-0.73) | **< 0.001** | 0.9  (0.82-0.99) | **0.029** |
| **Obesity** | 1.0 (0.61-1.64) | 0.985 | 0.97 (0.74-1.28) | 0.830 | 0.99 (0.72-1.35) | 0.934 | 1.02 (0.86-1.21) | 0.828 |
| **Dyslipidemia** | 0.61 (0.42-0.88) | **0.007** | 1.03 (0.85-1.25) | 0.739 | 0.65 (0.52-0.81) | **< 0.001** | 0.83 (0.74-0.94) | **0.002** |
| **Smoking** | 1.5 (0.91-2.46) | 0.113 | 0.82 (0.59-1.15) | 0.255 | 1.0 (0.71-1.4) | 0.983 | 0.81 (0.67-0.98) | **0.027** |
| **Diabetes** | 0.57 (0.43-0.76) | **< 0.001** | 1.67 (1.42-1.97) | **< 0.001** | 0.70 (0.6-0.83) | **< 0.001** | 1.53 (1.4-1.67) | **< 0.001** |
| **CAD** | 1.6 (1.2-2.1) | **0.001** | 0.97 (0.8–1.17) | 0.728 | 1.55 (1.31.85) | **< 0.001** | 0.93 (0.84-1.04) | 0.189 |
| **CHF** | 4.78 (3.62-6.31) | **< 0.001** | 1.07 (0.85-1.33) | 0.575 | 3.65 (3.08-4.32) | **< 0.001** | 1.17 (1.04-1.31) | **0.010** |
| **CKD** | 2.01 (1.53-2.67) | **< 0.001** | 1.24 (1.04-1.47) | **0.017** | 1.61 (1.30-1.9) | **< 0.001** | 0.91 (0.82-0.99) | **0.044** |
| **Malignancies** | 4.0 (2.4-6.69) | **< 0.001** | 1.91 (1.26-2.91) | **0.002** | 3.27 (2.39-4.47) | **< 0.001** | 0.89 (0.7-1.14) | 0.350 |

OR - Odds Ratio; CI - confidence interval; CAD - coronary artery disease; CHF - chronic heart failure; CKD - chronic kidney disease.

Binary logistic regression analysis for the end-points in-hospital mortality and in-hospital amputation in patients at Rutherford grade 5 and Rutherford grade 6. In the logistic regression model, effects of included variables are presented as Odds Ratio and corresponding confidence intervals, p-values <0.05 are considered significant.
